# Effect of feed restriction on dairy cow milk production: a review

**DOI:** 10.1093/jas/skab130

**Published:** 2021-07-01

**Authors:** Antoine Leduc, Sylvain Souchet, Marine Gelé, Fabienne Le Provost, Marion Boutinaud

**Affiliations:** 1 Institut Agro, INRAE, PEGASE, 35590 Saint Gilles, France; 2 Université Paris-Saclay, INRAE, AgroParisTech, GABI, 78350 Jouy-en-Josas, France; 3 Institut de l’Elevage, 49105 Angers, France

**Keywords:** dairy cow, feed restriction, mammary gland, metabolism, milk

## Abstract

In the dairy cow, negative energy balance affects milk yield and composition as well as animal health. Studying the effects of negative energy balance on dairy cow milk production is thus essential. Feed restriction (FR) experiments attempting to reproduce negative energy balance by reducing the quantity or quality of the diet were conducted in order to better describe the animal physiology changes. The study of FR is also of interest since with climate change issues, cows may be increasingly faced with periods of drought leading to a shortage of forages. The aim of this article is to review the effects of FR during lactation in dairy cows to obtain a better understanding of metabolism changes and how it affects mammary gland activity and milk production and composition. A total of 41 papers studying FR in lactating cows were used to investigate physiological changes induced by these protocols. FR protocols affect the entire animal metabolism as indicated by changes in blood metabolites such as a decrease in glucose concentration and an increase in non-esterified fatty acid or β-hydroxybutyrate concentrations; hormonal regulations such as a decrease in insulin and insulin-like growth factor I or an increase in growth hormone concentrations. These variations indicated a mobilization of body reserve in most studies. FR also affects mammary gland activity through changes in gene expression and could affect mammary cell turnover through cell apoptosis, cell proliferation, and exfoliation of mammary epithelial cells into milk. Because of modifications of the mammary gland and general metabolism, FR decreases milk production and can affect milk composition with decreased lactose and protein concentrations and increased fat concentration. These effects, however, can vary widely depending on the type of restriction, its duration and intensity, or the stage of lactation in which it takes place. Finally, to avoid yield loss and metabolic disorders, it is important to identify reliable biomarkers to monitor energy balance.

## Introduction

Dairy cows are highly susceptible to being in negative energy balance. In dairy cow, feed restriction (FR) can lead to a negative energy balance state. This state is reached by an animal when the energy brought by its food supply is lower than its energy needs. This state can occur physiologically, as with early lactation, or it can be environmentally induced, as in cases of food shortage. During late pregnancy and early lactation, cows have a decreased voluntary feed intake, which may be the result of physical constraints, nervous, and hormonal signals (Ingvartsen et al., 1999). This intake reduction, coupled with the high-energy needs of lactation establishment, often leads to negative energy balance that lasts until the first weeks of lactation. Indeed, the beginning of lactation is characterized by increasing energy needs linked to the rapid increase in mild yield, which leads to negative energy balance, body reserve mobilization, and milk composition modification (Bjerre-Harpøth et al., 2012). This key period, during which lactation is established, requires special attention to avoid metabolic disorders that could affect the whole lactation. Moreover, in the current climate change context, drought periods may become increasingly common and forage yield and quality may be affected (Godde et al., 2019). Grazing systems are highly sensitive to drought periods that affect grass growth and quality, leading to feed shortages, reducing energy and protein input, and thus affecting milk production (Lemaire and Pflimlin, 2007). Thus, it is important to understand the mechanisms of metabolic adaptation to FR to avoid related problems. Experiments of FR attempt to mimic negative energy balance occurring naturally, whether physiologically at the beginning of lactation or environmentally during food shortage. In most publications (Tables 1 to 3), FR is induced during mid-lactation which is more convenient to run experimental protocols (Billa et al., 2020). References in other species including other ruminants are also cited to support what is observed in cows or in the case of lack of data in cattle. Indeed, physiological changes observed with FR are mostly commonly observed in mammals.

**Table 1.** Effects of FR on concentrations in plasma of indicators of body reserve mobilization in lactating dairy cows, variations are expressed as a percentage of the control value

Restriction type	DMI^1^	Duration, d	Day in milk	NEFA^2^	BHB^3^	Glucose	Insulin	Animal number	Reference
Qualitative	−58%	5	329 ± 12	+826%	ns^4^	−13%	− ^5^	21	Ollier et al. (2015)
Qualitative	−56%	4	25 ± 5	+160%	+320%	−31%	−30%	17	Pires et al. (2019)
Qualitative	−55%	21	98 ± 7	+81%	+33%	−5%	−	50	Gross et al. (2011a)
Qualitative	−44%	4	Multiple	+34%	+175%	−11%	−79%	47	Bjerre-Harpøth et al. (2012)*
Qualitative	−16%	320	−17	+32%	−	−6%	−	352	Delaby et al. (2009)
Qualitative	−	77	−14	+139%	−	−	−	16	Dessauge et al. (2011)
Qualitative	−	2.5	98 ± 18	+1057%	−	ns	−	16	Kuhla et al. (2010)
Qualitative	ns	56	1	ns	+26%	−8%	−46%	40	Andersen et al. (2003; 2004)
Quantitative	−100%	6	30	+500%	−	−24%	−	10	Reid et al. (1977)
Quantitative	−100%	2	69 ± 9	+3475%	ns	−23%	−86%	12	Agenäs et al. (2003)*
Quantitative	−100%	2	55 ± 8	+525%	−	−18%	−56%	11	Chelikani et al. (2004)
Quantitative	−100%	2	175 ± 3	+1200%	−	−27%	−88%	4	McGuire et al. (1995
Quantitative	−100%	1	45 ± 2	+319%	ns	−25%	−60%	3	Toerien andand Cant 2007)*
Quantitative	−83%	5	157 ± 9	+274%	ns	ns	−83%	5	Kvidera et al. (2017)
Quantitative	−60%	5	157 ± 9	+175%	ns	ns	−76%	5	Kvidera et al. (2017)
Quantitative	−41%	5	157 ± 9	ns	ns	ns	−77%	5	Kvidera et al. (2017)
Quantitative	−22%	5	157 ± 9	ns	ns	ns	−68%	5	Kvidera et al. (2017)
Quantitative	−64%	7	77 ± 12	+215%	+57%	−24%	−47%	10	Moyes et al. (2009)
Quantitative	−64%	6	165 ± 21	+527%	+5%	−9%	−58%	10	Billa et al. (2020)*
Quantitative	−50%	5	Multiple	+215%	ns	ns	−47%	8	Carlson et al. (2006)
Quantitative	−47%	4	223 ± 103	+306%	−	ns	ns	13	Contreras et al. (2016)
Quantitative	−47%	4	204 ± 29	+129%	−	−7%	−50%	8	Ferraretto et al. (2014)
Quantitative	−43%	5	156 ± 6	+14%	−	ns	ns	16	Velez andand Donkin 2005)
Quantitative	−43%	4	91 ± 5	+120%	+74%	ns	−	7	Laeger et al. (2012)
Quantitative	−40%	21	34 ± 6	+153%	+173%	−14%	−31%	120	Kay et al. (2013)
Quantitative	−40%	14	97 ± 11	+448%	−	−	−	24	Perkins et al. (2002)
Quantitative	−39%	4	84 ± 17	+500%	−	−	−	10	Abdelatty et al. (2017)
Quantitative	−38%	63	70 ± 7	ns	−	ns	−	8	Vicini et al. (1988)
Quantitative	−37%	29	14	+97%	+108%	ns	−8%	16	Drackley et al. (1991)
Quantitative	−35%	14	1	+158%	+42%	ns	ns	11	Radcliff et al. (2006)
Quantitative	−34%	3	35 ± 8	+121%	+90%	−19%	−	8	Nielsen et al. (2003)
Quantitative	−31%	29	14	+162%	+195%	ns	ns	13	Drackley et al. (1992)
Quantitative	−30%	30	14	+206%	+721%	−22%	ns	18	Veenhuizen et al. (1991)
Quantitative	−28%	20	159 ± 40	+86%	−	ns	ns	24	Lapierre et al. (1995)
Quantitative	−24%	6	132 ± 8	+180%	−	−	−	12	Capuco et al. (2001)
Quantitative	−13%	21	88 ± 17	+97%	ns	−3%	−	16	Vanbergue et al. (2018)
Quantitative	−21%	21	88 ± 17	+102%	+31%	ns	−	16	Vanbergue et al. (2018)
Quantitative	−20%	29	77 ± 5	+355%	−	ns	−36%	19	Herve et al. (2019)

^1^DMI, dry matter intake.

^2^NEFA, non-esterified fatty acid.

^3^BHB, β-hydroxybutyrate.

^4^ ns, notsignificant (*P*-value > 0.05).

^5^: –, no data available.

*: No control group available in these studies, percentages are calculated relatively to the pre-experimental values.

To induce FR, 2 different types of protocols are primarily carried out: quantitative or qualitative FR. Quantitative FR provides a controlled amount of feed per animal, calculated as a percentage of ad libitum dry matter intake (DMI) during the pre-experimental period. Qualitative FR uses low-energy or low-protein diets. This restriction can be achieved by changing the proportion of different ingredients in the ration to decrease the nutrient density, by removing some or all concentrate or by diluting the diet with nondigestible high-fiber feed components, such as hay. Among the references used in this review, 41 papers focus on FR in lactating dairy cows. Three-quarters of these studies used quantitative protocols with FR intensity ranging from as low as −20% of DMI to total feed deprivation (−100% of DMI). In this review, FR are considered as severe when DMI is reduced by more than 50%, these protocols were generally short, with a duration of less than a week. Variations induced by FR protocols are expressed as percentages of the control group values if available, or of pre-experimental values if not. Statistical analyses are specific of each paper but a significance threshold of 0.05 was adopted for all.

The aim of this article is to review the results of FR experiments performed during lactation in dairy cows to characterize metabolic changes occurring during these challenges and to describe how these changes affect mammary gland activity and milk production and composition.

## Effect of FR on Animal Physiology

### Endocrine adaptation

**Figure 1.**
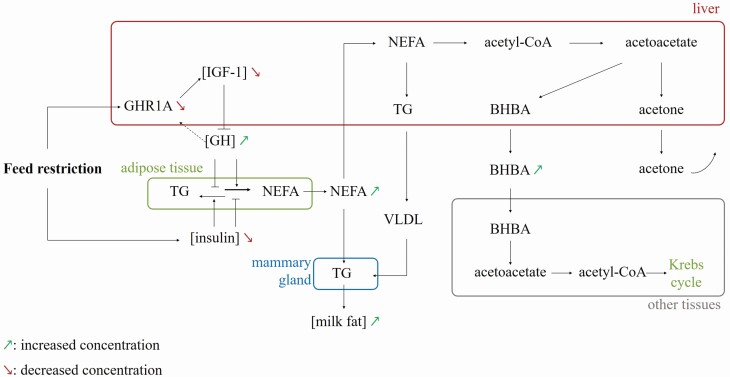
Proposition of a schematic representation of the metabolism of body reserve mobilization that can take place during negative energy balance. FR is able to decrease insulin concentration and, by downregulating the expression of liver growth hormone receptor (GHR1A), to decrease insulin-like growth factor I (IGF-1) concentrations. As IGF-1 negative feedback is less active, the GH concentration increases. High GH and low insulin concentrations in plasma enhance triglyceride (TG) degradation into NEFA in adipose tissue. In the liver, NEFA can be used to produce TG or acetyl-CoA. TG in the form of very low density lipoprotein (VLDL) and NEFA can be used by the mammary gland to produce milk fat. When glucose concentrations are low in the liver, acetyl-CoA is used for ketogenesis, rather than for the Krebs cycle. Acetoacetate is formed and can either be decarboxylated into acetone and then excreted or reduced into BHBA. Other tissues in need of energy, such as the brain, skeletal muscle, or heart, can use BHBA to synthesize acetyl-CoA and to produce energy via the Krebs cycle.

Experiments of FR lead to a decreased insulin concentration in plasma in 18 out of 24 studies that measured this hormone. When significant, these decreases vary from −8% to −88% and seem to be more elevated when FR is more intense (Table 1). When DMI is reduced by more than 50%, insulinemia is always reduced as shown in 10 experiments from Table 1. When DMI is reduced by less than 50%, only half of the studies reported a reduction in insulinemia (6 out of 12). Only 1 out of 6 studies found a significant variation of glucagon during FR with a −30% decrease (Vicini et al., 1988). Insulin is a major lipogenesis regulator and is known to enhance protein synthesis. Insulin also affects the growth hormone (GH) signaling pathway by regulating the expression of GH receptors (GHR). There are 3 types of GHR: GHR 1A, primarily expressed in the liver, and GHR 1B/1C in other tissues especially in adipocytes. Only GHR 1A is under nutritional regulation (Lucy et al., 2001). Insulin has different effects in liver and adipose tissue. It stimulates the expression of GHR 1A in the liver, whereas it inhibits the expression of GHR 1B/1C in adipose tissue (Butler et al., 2003). When the insulin concentration in plasma is low, the GHR 1A concentration decreases in the liver. Nevertheless, insulin may not be the only cause for GHR 1A underexpression. Radcliff et al. (2006) showed that during the postpartum period insulin concentration starts to decrease 2 or 3 d after the decrease in GHR 1A. The binding of GH to GHR 1A regulates insulin-like growth factor I (IGF-1) synthesis; IGF-1 is thus less expressed when the GHR 1A concentration is low (Fenwick et al., 2008). The decrease in IGF-I concentration in plasma decreases the negative feedback on GH, whose concentration in plasma increases (Figure 1). Thus the concentration of GH in plasma was significantly increased in 6 out of 9 studies with variation that ranged from +64% to +450% (Table 2). The concentration of IGF-1 in plasma was significantly decreased in 7 out of 10 studies with variations that ranged from −13% to −49% (Table 2). Concentrations of both GH and IGF-1 varied inversely significantly in 4 out of 7 studies, which included the 2 fasting studies that measured these hormone concentrations (Table 2). Greater GH concentrations enhance gluconeogenesis in the liver and lipolysis in adipose tissue. The decreased concentration of IGF-1 paired with increased concentration of GH observed during intense FR corresponds to the uncoupling of the GH–IGF-1 axis that also occurs under negative energy balance at the beginning of lactation (Lucy, et al., 2001, Keogh et al., 2015). An insulin resistant state could also take place during negative energy balance as high concentrations of GH and non-esterified fatty acids (NEFA) antagonize the insulin pathway (Bell and Bauman, 1997; Lucy, 2004). Short and intense FR can induce peaks in cortisol concentrations that range from +221% to +648%, probably to allow milk production conservation (Agenäs et al., 2003; Toerien and Cant, 2007; Moyes et al., 2009). Nevertheless, Herve et al. (2019) showed that a moderate and longer restriction can induce a decrease (−26%) in cortisol concentration after milking without any variation of basal concentration, and Pires et al. (2019) did not see any significant variation with a shorter and more intense restriction. Experiments of FR can also decrease the concentration of prolactin in plasma, which stimulates milk production, from −38% to −86% (Vicini et al., 1988; Kuhla et al., 2010; Ollier et al., 2015). Nevertheless, Herve et al. (2019) did not observe any significant prolactin concentration variation during their moderate restriction protocol. During short-term feed deprivation, Chelikani et al. (2004) also described a decreased concentration of leptin in plasma (−39%). This reduction of leptin expression could be mediated by insulin (Saremi et al., 2014). Leptin is primarily secreted by adipocytes and is involved in the regulation of ingestion, fat metabolism, energy balance, insulin sensitivity, and appetite (Reist et al., 2003). Ferraretto et al. (2014) described an increase in the concentration of progesterone (+14%). No significant variation in concentration in plasma was found for FSH (Ferraretto et al., 2014) and LH (Kuhla et al., 2010).

**Table 2.** Effects of FR on concentrations in plasma of GH and insulin-like growth factor (IGF)-I in lactating dairy cows, variations are expressed as a percentage of the control value

Restriction type	DMI^1^	Duration, d	Day in milk	GH^2^	IGF-1^3^	Animal number	Reference
Qualitative	− ^4^	77	−14	+310%	−34%	16	Dessauge et al. (2011)
Qualitative	−	2.5	98 ± 18	ns^5^	−	16	Kuhla et al. (2010)
Qualitative	ns	56	1	+111%	−37%	40	Andersen et al. (2003 2004)
Quantitative	−100%	2	55 ± 8	+450%	−39%	11	Chelikani et al. (2004)
Quantitative	−100%	2	175 ± 3	+85%	−49%	4	McGuire et al. (1995)
Quantitative	−40%	21	34 ± 6	ns	−33%	120	Kay et al. (2013)
Quantitative	−38%	63	70 ± 7	+87%	−	8	Vicini et al. (1988)
Quantitative	−35%	14	1	ns	−31%	11	Radcliff et al. (2006)
Quantitative	−28%	20	159 ± 40	+64%	ns	24	Lapierre et al. (1995)
Quantitative	−25%	21	162 ± 20	−	ns	5	Guinard-Flament et al. (2007)
Quantitative	−24%	6	132 ± 8	−	ns	12	Capuco et al. (2001)
Quantitative	−20%	29	77 ± 5	−	−13%	19	Herve et al. (2019)

^1^DMI, dry matter intake.

^2^GH, growth hormone.

^3^IGF-1, insulin-like growth factor-1.

^4^–, no data available.

^5^ns, not-significant (*P*-value > 0.05).

After the FR period, most endocrine factors quickly return to initial concentrations (Chelikani et al., 2004; Pires et al., 2019). Nevertheless, the concentration of insulin in plasma, which is decreased during FR, can briefly spike after refeeding before its return to control values (Agenäs et al., 2003; Bjerre-Harpøth et al., 2012). Interestingly, basal cortisol and prolactin concentrations (before milking) were shown to be lower in cows switched back to an ad libitum feeding compared with cows that were always fed ad libitum diet (Herve et al., 2019). In contrast, the prolactin concentration after milking was higher suggesting a potential adaptive role of prolactin to maintain lactation after a period of FR (Herve et al., 2019).

To conclude, FR affects the organism through multiple hormonal regulations. Insulin, IGF-1, leptin, glucagon, and prolactin concentrations can be decreased, whereas GH, progesterone, and cortisol concentrations can be increased, especially when FR is intense. In fact, insulin, GH, and IGF-1 are always affected during severe FR studies which is not the case in half of less intense FR studies (Tables 1 and 2). Taken together, these modifications enhance body reserve mobilization through lipolysis and gluconeogenesis and redirect nutrients to vital organs. Similar adaptations are observed during early lactation negative energy balance (Smith et al., 1976; van Knegsel et al., 2007).

### Blood metabolites

In connection with the decreased insulin concentration in plasma, FR led to a decreased glucose concentration in plasma in 18 out of 34 studies that measured this metabolite. Significant variations ranged from 5% to -31%, and were mainly observed during fasting experiments and severe restrictions (Table 1). When glycemia was affected, its variation seems to be more elevated when FR is more intense (Table 1). In the case of moderate FR (with DMI reduction ≤ 50%), glycemia was stable in 14 out of 21 studies. For a low FR level (approximately −20% DMI), the lack of effect of FR on glycemia could depend on the type of diet, since its variation was shown to be significant with a corn-based FR diet and did not vary with a grass-based FR diet (Vanbergue et al., 2018). High GH and low insulin concentrations in plasma are known to promote body reserve mobilization through lipolysis (Stipanuk, 2000). Adipose tissue is the main energy reserve of the body; it contains adipocytes that are full of triglycerides (Bell, 1995). In adipocytes, there is a constant balance between lipogenesis and lipolysis (Figure 1). Lipolysis results in the production of NEFA that are released in blood circulation. The concentration of NEFA in plasma was significantly increased in 34 out of 38 studies. When significant, these variations ranged from +14% to +3475%, with fasting and severe diet dilution leading to the highest increases (Table 1). NEFA can be directly used by the mammary gland as a source of milk fat, re-esterified into triglycerides in the liver or β-oxidized in the liver (Drackley, 1999). Triglycerides are normally released in blood as very low density lipoproteins, but this process is slow; therefore, an accumulation of triglycerides in the liver can occur, which causes a metabolic disorder called hepatic steatosis, or “fatty liver”. Increased concentrations of triglycerides (Veenhuizen et al., 1991; Moyes et al., 2009) and cholesterol (Reid et al., 1977; Moyes et al., 2009; Laeger et al., 2012) in plasma can also appear during FR, but are not always significant (Drackley et al., 1992; Bjerre-Harpøth et al., 2012). In liver mitochondria, NEFA, after being β-oxidized in acetyl-CoA, can be either fully oxidized in the Krebs cycle or partially oxidized into ketone bodies. During FR, NEFA are primarily used for ketogenesis because of a slowdown of the Krebs cycle. This slowdown is caused by an inhibition of isocitrate and α-ketoglutarate dehydrogenases and by a preferential utilization of oxaloacetate in the gluconeogenesis pathway (Herdt, 2000). Ketogenesis enzymes use 2 acetyl-CoA to produce acetoacetate, the first ketone body. Then, acetoacetate can either be decarboxylated in acetone or reduced in β-hydroxybutyrate (BHBA). These 3 ketone bodies are released in blood circulation and can be excreted by kidneys for acids or by lungs and milk for acetone. Tissues where the Krebs cycle is not slowdown can oxidize BHBA in acetoacetate and use it to resynthesize acetyl-CoA for Krebs cycle use (Bergman, 1971). Concentration of BHBA was significantly increased in 14 out of 23 studies with variation ranging from +26% to +721% when significant (Table 1). Parameters controlling the intensity of body reserve mobilization at the beginning of lactation have been extensively studied. Genetic parameters such as milk yield potential (Daniel et al. 2018), body condition score (BCS; Pires et al. 2013), catecholamines or the number of adrenergic receptor in adipose tissue (Weber et al. 2013) are known to affect body reserve mobilization at early lactation and could intervene in the variation in BHBA concentration during FR. However, few studies have been done during FR. The collected data suggest that the intensity of the BHBA reaction to FR depends on the lactation stage, with the highest variations occurring in early lactation (Veenhuizen et al., 1991; Pires et al., 2019) and on the type of diet, being significant with a corn-based FR diet and not varying with a grass-based FR diet (Vanbergue et al., 2018). An accumulation of ketone bodies can lead to a common disease called ketosis or acetonemia. This disease is characterized by high blood, urine, and milk concentrations of ketone bodies, and its clinical state causes decreased appetite, weight loss, and decreased milk yield. A rarer nervous form can occur if clinical ketosis is coupled with important hypoglycemia (Scott et al., 2011). Even if most studies tried to avoid it for welfare reason, clinical ketosis can occur under FR (Veenhuizen et al., 1991). An increase in acetate concentration in plasma (+364%, +136%, and +20%, respectively) has been described (Veenhuizen et al., 1991; Toerien and Cant, 2007; Vanbergue et al., 2018) but was not significant by Drackley et al. (1991; 1992) and was not shown by Guinard-Flament et al. (2007). These difference of effect of FR on acetate concentration may be linked to the lactation stage, with increases being observed only in early lactation. Veenhuizen et al. (1991) also described a decrease in glycogen concentration in plasma (−90%). The α amino acid concentration did not seem to vary under FR (McGuire et al., 1989; Guinard-Flament et al. 2007; Toerien and Cant 2007). Agenäs et al. (2003) and Pires et al. (2019) observed an increased urea concentration (+40% and +33%, respectively) while Delaby et al. (2009) observed a decreased urea concentration (−21%) with a softer restriction protocol. Laeger et al. (2012), Herve et al. (2019), and Vanbergue et al. (2018), on their side, did not observe any significant variation of urea concentration in plasma. Increased concentrations of urea in plasma during high intensity FR and fasting protocols can be a result of amino acid catabolism in order to produce energy to compensate low-energy intake. In contrast, decreased concentration of urea in plasma could be linked to a diet dilution with a decreased proportion of protein concentrate in the diet. This decreased nitrogen intake leads to a decreased production of urea in the rumen and could result in a decreased urea concentration in plasma. Softer restriction protocols without modification of nitrogen:energy ratio in diets did not affect urea concentration in plasma. Similarly, Kvidera et al. (2017) showed that plasma urea nitrogen reaction seems to vary with FR intensity: soft and moderate restrictions (−20% and −40% of DMI) lead to −20% and −29% decreases in concentration of urea nitrogen whereas severe restrictions (−60% and −80% of DMI) lead to −51% and −49% decreases in concentration of urea nitrogen. This is confirmed by Velez and Donkin (2005) with a −19% decrease in concentration of urea nitrogen in plasma during a moderate restriction protocol (−43% of DMI). Nevertheless, Andersen et al. (2004) showed a slight increased concentration (+3%) with a soft diet dilution protocol (−19% of net energy in diet) and other studies did not observe any significant variation in plasma urea nitrogen (McGuire et al., 1989; Toerien and Cant 2007; Bjerre-Harpøth et al., 2012).

After the FR period, glucose concentrations in plasma quickly return to control concentrations (Chelikani et al., 2004; Herve et al. 2019; Pires et al., 2019). Similarly, concentrations of BHBA, NEFA, triglycerides, and cholesterol return to normal within 1 to 14 d (Reid et al., 1977; Bjerre-Harpøth et al., 2012; Pires et al., 2019). Nevertheless, after refeeding, concentrations of glucose in plasma can briefly spike (Bjerre-Harpøth et al., 2012; Reid et al., 1977) and concentrations in plasma of BHBA (Agenäs et al., 2003), triglycerides and cholesterol (Reid et al., 1977) can briefly dip before their return to control values. For glucose, it can be the result of an overcompensation of homeostatic mechanisms whereas for lipids it can be the result of an increased uptake by mammary and peripheral tissues (Reid et al., 1977).

Thus, FR enhances lipolysis over lipogenesis, which produces high quantities of NEFA that are metabolized into ketone bodies to provide energy to tissues such as brain, heart, skeletal muscles or mammary gland in which glucose is preferably oriented in early lactation.

### Changes in body composition, heart rate, and respiration rate

Among the references used, only 19 papers showed an effect of FR on the energy balance, which was always negative, ranging from −1.5 to −24.9 Mcal/d. This negative energy balance induced by FR leads to a loss of body weight. This loss of body weight is linked to decreased DMI, loss of gut fill and somewhat to body reserve mobilization (Roche et al., 2009; Gross et al., 2011b). When measured, body weight was significantly decreased in 17 out of 20 studies, with variations ranging from −4% to −13%. Nevertheless, body weight does not accurately reflect reserve mobilization as water partially replaces fat in tissues (Schröder and Staufenbiel, 2006). Body reserve mobilization is evaluated using the determination of the BCS, a visual and tactile notation that evaluates the subcutaneous fat layer (Wildman et al., 1982; Ferguson et al., 1994). A decrease in BCS can be induced by FR (Friggens et al., 1998; Delaby et al., 2009; Gross et al., 2011a; Ferraretto et al., 2014) which is not always significant compared with the control group (Chelikani et al., 2004; Pires et al., 2019). Dessauge et al. (2011) also described a loss of mammary gland weight during a 13-wk severe diet dilution, whereas Nørgaard et al. (2005) did not see any significant mammary gland size change during a 16-wk low-energy density diet. Mammary weight loss may only happen in the most severe FR, when apoptosis and mammary gland remodeling take place. This has been observed only at the beginning of lactation (Dessauge et al. 2011).

Moyes et al. (2009) noticed heart and respiration rates slowdown during short-term severe FR (−30%) but no significant effect on rectal temperature. Similarly, Kvidera et al. (2017), who designed a study with groups of cows exposed to 20%, 40%, 60%, or 80% FR for 5 d, showed that heart and respiration rates declined linearly with FR, but did not observe any significant modification of rectal temperature. McGuire et al. (1989) did not observe any significant effect on heart and respiration rates or on rectal temperature with a more moderate restriction protocol.

## Effect of FR on the Mammary Gland

Under FR, milk production and corresponding mammary metabolism are decreased. IGF-1, whose concentration in plasma is generally decreased under FR, is known to stimulate mammary blood flow and milk secretion (Prosser et al., 1990). Guinard-Flament et al. (2007) showed decreased mammary blood flow along with significant reductions in mammary nutrient uptake (glucose, acetate, BHBA, glycerol, and α-amino nitrogen) as well as diminutions in dioxygen uptake and carbon dioxide output for dairy cows under 30% FR.

These modifications are associated with changes in the gene expression profile in the mammary gland. Dessauge et al. (2011), during long and intense FR, showed a decreased expression of *LALBA* and *CSN3*, which encode 2 major milk proteins, without affecting *CSN1S1* gene expression (Beaujean et al., 2020). Such differences in gene expression response to FR between caseins have also been observed in goats (Tsiplakou et al., 2016) and sheep (Tsiplakou et al., 2015a). Nevertheless Boutinaud et al. (2008) and Herve et al. (2019) did not find any significant expression differences for these genes in cows under a more moderate restriction protocol. Boutinaud et al. (2008) revealed a downregulation of *SLC2A1*, a major glucose transporter analyzed in mammary epithelial cell (MEC) isolated and purified from milk. This effect of FR has also been shown in mammary gland in sheep (Tsiplakou et al., 2015b). Nevertheless, *SLC2A1* was not significantly impacted in milk purified MEC in cows during a slightly more moderate restriction experiment (Herve et al., 2019). Abdelatty et al. (2017) also found downregulation of several mammary lipogenic genes during a 4-d-long 40% FR protocol: *ACACA*, *GPAM*, *SCD1*, *FABP3*, *LPL*, and *SREBF1*. Finally, Dessauge et al. (2011) also showed an upregulation of apoptosis genes: *BAD*, *PTEN*, *CASP3*, *CTSB*, *IGFBP5*, and *CAPN2*. Nevertheless, during a less severe FR, Herve et al. (2019) did not find that *CASP3* was significantly impacted. In goats, Ollier et al. (2007) performed a mammary transcriptomic analysis after a 2-d-feed deprivation that showed downregulation of 141 genes among which genes involved in proliferation, differentiation as well as milk protein, lactose, and lipid metabolism. Only 20 genes were upregulated during this experiment. These results showed a stress response by the mammary gland and a slowdown of MEC activity during this short feed deprivation.

Moreover, Singh et al. (2012) suggested that nutrition could induce epigenetic mechanisms such as DNA methylation and thus regulate milk production for subsequent lactation cycles and even for subsequent generations. In a study where FR induced a 38% drop in milk production (Dessauge et al., 2011), a trend toward higher global DNA methylation in the mammary tissue was observed (Beaujean et al., 2020). Nevertheless no variation in the percentage of DNA methylation in the distal region upstream *CSN1S1* gene. Further research is needed to elucidate if epigenetic modifications could be involved in gene expression changes induced by FR.

Protocols of FR can also impact microRNA (miRNA) abundance, as shown by Mobuchon et al. (2015) in goat mammary glands. miRNA is small noncoding RNA involved in the posttranscriptional regulation of gene expression (Bartel, 2004). Mobuchon et al. (2015) was the first miRNome study on feed restricted lactating ruminants. Similar analyses of the mammary gland has been recently reported (Billa et al. 2021). In 48 hr food-deprived goats, Mobuchon et al. (2015) found 30 nutriregulated miRNA, the prediction of targeted mRNA revealed that “gene expression,” “cellular development,” and “cellular growth and proliferation” were the most significantly targeted pathways and that some of these miRNA may regulate milk lipid and protein synthesis. A recent study performed in cow showed that FR affected 8 miRNA and 374 differentially expressed mRNAs mainly involved in lipid metabolism and endothelial cell proliferation confirming resuls observed in goats (Billa et al. 2021).

Dessauge et al. (2011) suggested that FR could lead to MEC apoptosis and mammary gland involution through activation of matrix metallopeptidases (MMP2 and MMP9). In fact, in vitro experiments have shown that, when insulin and IGF-1 signaling decrease, a degradation of the extracellular matrix induced by matrix metallopeptidases promotes apoptosis of MEC (Alexander et al., 1996; Farrelly et al., 1999). Similar reactions have been described in mice during involution of the mammary gland (Talhouk et al., 1992). This finding is in keeping with the smaller acini, disorganized structure, lower total amount of DNA and lighter mammary glands observed after a long FR at early lactation (Dessauge et al., 2011). Nørgaard et al. (2005) found that MEC proliferation was considerably lower in cows fed a low-energy-density diet at 8 wk postpartum. However, the effect on cell proliferation was no longer observed at 16 wk postpartum (Nørgaard et al., 2005). Herve et al. (2019) showed that the decrease in milk yield associated with FR could also be attributable to an increase in MEC exfoliation. In this study, dairy cows were under a moderate intensity FR that did not lead to significant modification of mammary tissue organization, MEC proliferation, and apoptosis or gene expression. Nevertheless, this experiment led to a 65% increase in the MEC exfoliation rate, which is another way to decrease the number of MEC and thus to decrease milk yield (Herve et al., 2019). This exfoliation is also a sign of a loss of mammary epithelium integrity, which could lead to apoptosis and reduction of MEC activity (Ben Chedly et al., 2010).

To conclude, depending on its duration and intensity or the stage of lactation, FR can induce a slowdown of milk production metabolism, as well as a decrease in MEC number through higher exfoliation or can enhance gene regulation to anticipate involution of the mammary gland.

## Effect on Milk Production and Composition

FR usually significantly decreased milk yield (41 out of 44 studies), with variations ranging from −7% to −71% (Table 3). The highest milk yield decreases are observed during fasting and severe diet dilution. The decreased milk yields are also related to a reduction of mammary gland activity. Milk lactose content is also often decreased (19 out of 31 studies), with variations ranging from −2% to −20% (Table 3). One study showed that lactose content was decreased by a grass-based restriction diet but was not affected by a corn-based restriction diet (Vanbergue et al., 2018). This finding suggests that the type of diet could influence the effect of FR on lactose content. Decreased lactose contents and yields could probably be induced by lower glycemia and decreased glucose uptake by the mammary gland. A link between glycemia and lactose content is also supported by the fact that the decreased lactose content induced by a grass-based restriction diet is accompanied with a decreased glycemia while, the corn-based restriction diet did not affect either lactose content or glycemia (Vanbergue et al., 2018). Decreased lactose synthesis may also be a consequence of the downregulation of such genes as *LALBA* and *SLC2A1, LALBA*, which code for the co-factor of the enzyme responsible for lactose synthesis and *SLC2A1*, which codes for the transporter of the main lactose precursor. Milk protein and fat contents in milk are also sometimes impacted. Protein content may be decreased (19 out of 36 studies) with variations ranging from −3% to −17% (Table 3), nevertheless increased protein contents have also been observed (2 out of 36 studies; McGuire et al., 1995; Lacy-Hulbert et al., 1999; Table 3). The higher protein content could be the result of a higher serum protein content in milk in relation to an integrity loss of the mammary epithelium in case of severe FR (Lacy-Hulbert et al., 1999). Milk fat content can be increased (18 out of 38 studies) with variations that range from +6% to +129% and with severe diet dilutions leading to the highest increases (Table 3). The increase in fat content is due to long chain fatty acids coming from lipomobilization that compensates for the decrease in de novo fatty acid synthesis (Abdelatty et al., 2017; Vanbergue et al., 2018; Billa et al., 2020). Protein content seems to be primarily impacted when low-energy or low-protein diets are used, whereas fat content is more significantly impacted under severe restriction. Kvidera et al. (2017) observed a linear decrease in both milk yield and energy balance after exposing cows to 20%, 40%, 60%, or 80% FR for 5 d. In this study, advanced levels of FR also induced a linear increase in milk fat content and somatic cell count and a linear decrease in milk protein and lactose contents, demonstrating clearly that the variation of milk composition depends on the FR intensity. Experiments of FR can also induce an increase in sodium concentration and somatic cell count in milk (Lacy-Hulbert et al., 1999; Herve et al., 2019) which reflects the loss of mammary epithelium integrity.

**Table 3.** Effects of FR on milk yield and concentrations of major milk constituent in lactating dairy cows, variations are expressed as a percentage of the control value

Restriction type	DMI^1^	Duration, d	Day in milk	Milk yield	Fat content	Protein content	Lactose content	Animal number	Reference
Qualitative	−58%	5	329 ± 12	−54%	− ^2^	−	−	21	Ollier et al. (2015)
Qualitative	−56%	4	25 ± 5	−39%	+54%	ns^3^	−7%	17	Pires et al. (2019)
Qualitative	−55%	21	98 ± 7	−10%	ns	−6%	ns	50	Gross et al. (2011a)
Qualitative	−44%	4	Multiple	−35%	+42%	−9%	−5%	47	Bjerre-Harpøth et al. (2012)*
Qualitative	−26%	182	11 ± 5	−28%	+12%	−5%	−3%	24	Friggens et al. (1998)
Qualitative	−16%	320	−17	−18%	−1%	−4%	−	352	Delaby et al. (2009)
Qualitative	−	112	1	−22%	+50%	−	−2%	20	Nørgaard et al. (2005)
Qualitative	−	77	−14	−38%	ns	−10%	−4%	16	Dessauge et al. (2011)
Qualitative	ns	56	1	−18%	+17%	−6%	−2%	40	Andersen et al. (2003; 2004)
Qualitative	−	14	−	−18%	ns	−8%	−	770	Burke et al. (2010)
Quantitative	−100%	6	30	−71%	+114%	−	−12%	10	Reid et al. (1977)
Quantitative	−100%	2	69 ± 9	−51%	+129%	ns	−15%	12	Agenäs et al. (2003)*
Quantitative	−100%	2	55 ± 8	−56%	+81%	ns	−18%	11	Chelikani et al. (2004)
Quantitative	−100%	2	175 ± 3	−66%	+82%	+24%	−	4	McGuire et al. (1995)
Quantitative	−100%	1	45 ± 2	−44%	ns	ns	−8%	3	Toerien andand Cant 2007)*
Quantitative	−83%	5	157 ± 9	−55%	+38%	−9%	−13%	5	Kvidera et al. (2017)
Quantitative	−60%	5	157 ± 9	−33%	13%	−9%	ns	5	Kvidera et al. (2017)
Quantitative	−41%	5	157 ± 9	−27%	ns	ns	ns	5	Kvidera et al. (2017)
Quantitative	−22%	5	157 ± 9	ns	ns	ns	ns	5	Kvidera et al. (2017)
Quantitative	−64%	7	77 ± 12	−19%	ns	−17%	−20%	10	Moyes et al. (2009)
Quantitative	−64%	5	165 ± 21	−34%	+14%	ns	ns	10	Billa et al. (2020)*
Quantitative	−50%	30	Multiple	−41%	ns	−3%	ns	50	Gabbi et al. (2016)
Quantitative	−50%	26	228 ± 18	−36%	+9%	+8%	−2%	24	Lacy-Hulbert et al. (1999)
Quantitative	−50%	5	Multiple	−19%	ns	−7%	−4%	8	Carlson et al. (2006)
Quantitative	−47%	4	223 ± 103	−27%	ns	ns	−2%	13	Contreras et al. (2016)
Quantitative	−47%	4	204 ± 29	−23%	−	−	−	8	Ferraretto et al. (2014)
Quantitative	−43%	5	156 ± 6	−22%	ns	ns	−5%	16	Velez andandand Donkin 2005)
Quantitative	−43%	4	91 ± 5	−14%	−	−	−	7	Laeger et al. (2012)
Quantitative	−40%	21	34 ± 6	−28%	+14%	−10%	ns	120	Kay et al. (2013)
Quantitative	−39%	4	84 ± 17	−21%	ns	ns	−2%	10	Abdelatty et al. (2017)
Quantitative	−38%	63	70 ± 7	−18%	ns	ns	−	8	Vicini et al. (1988)
Quantitative	−37%	29	14	−7%	ns	ns	−	16	Drackley et al. (1991)
Quantitative	−35%	14	1	ns	−	−	−	11	Radcliff et al. (2006)
Quantitative	−34%	3	35 ± 8	−13%	+11%	ns	ns	8	Nielsen et al. (2003)
Quantitative	−31%	29	14	−20%	+21%	ns	−	13	Drackley et al. (1992)
Quantitative	−30%	30	14	−34%	−	−	−	18	Veenhuizen et al. (1991)
Quantitative	−28%	20	159 ± 40	−17%	+18%	−6%	−	24	Lapierre et al. (1995)
Quantitative	−25%	21	162 ± 20	−14%	ns	−8%	ns	5	Guinard-Flament et al. (2007)
Quantitative	−24%	8	96	−12%	−	−	−	6	McGuire et al. (1989)
Quantitative	−24%	6	132 ± 8	ns	ns	ns	−2%	12	Capuco et al. (2001)
Quantitative	−13%	21	88 ± 17	−12%	ns	−4%	ns	16	Vanbergue et al. (2018)
Quantitative	−21%	21	88 ± 17	−12%	ns	−4%	ns	16	Vanbergue et al. (2018)
Quantitative	−20%	29	77 ± 5	−9%	+6%	−5%	−2%	19	Herve et al. (2019)

^1^DMI, dry matter intake.

^2^—, no data available.

^3^ns, not-significant (*P*-value > 0.05).

*No control group available in these studies, percentages are calculated relatively to the pre-experimental values.

While milk, fat, and lactose yields recover within 2 d (Bjerre-Harpøth et al., 2012; Herve et al., 2019), protein yield seems to be slower to reach control values after refeeding (Herve et al., 2019).

There are few studies about milk minor constituents affected by FR. Larsen et al. (2016) designed an experiment in dairy cows in which protein density and digestibility of the ration varied and they measured minor milk constituents. These researchers found that lower total ration digestibility significantly decreased glucose-6-phosphate, glucose, and uric acid milk concentrations while significantly increasing BHBA, triacylglycerol, and urea milk concentrations. These modifications seem to reflect the variations of their concentrations in plasma. Moreover, lower protein density in the diet decreased glucose-6-phosphate and urea milk concentrations and increased BHBA, triacylglycerol, and cholesterol concentrations. Billa et al. (2020) recently described a decrease in BHBA, glucose, glutamate, uric acid, and free amino group concentrations in milk and an increase in glucose-6-phosphate and isocitrate concentrations in milk during a 6-d 50% FR in 2 different cow breeds. Increases in glucose-6-phosphate and isocitrate have also been reported in goats after 48 hr of feed deprivation (Chaiyabutr et al., 1981). Chaiyabutr et al. (1981) suggested that changes in minor milk constituent concentration can be explained by metabolic changes and disequilibrium between the use and the production of the metabolites occurring in the mammary gland. The elevation of some minor milk constituents could thus show evidence of metabolic blocking points. Bjerre-Harpøth et al. (2012) also identified citrate as the milk metabolite with the greatest increase during FR on cows.

To conclude, FR has a significant impact on milk production, with decreased milk yield and, depending on FR intensity, an increased milk fat content and somatic cell count as well as a decreased milk protein and lactose contents. When it comes to milk minor constituents, the results suggest that the direction of variation may differ depending on the nature of the restriction for some metabolites such as glucose-6-phosphate, BHBA, or urea, whereas others seem to be more reliable, such as glucose, uric acid, citrate, and isocitrate.

## Negative Energy Balance Biomarkers

As negative energy balance leads to lower milk yield and can induce metabolic disorders, such as ketosis or steatosis, it is important to have biomarkers able to monitor animals’ energy status. The interest of FR studies is to identify biomarkers allowing energy balance monitoring to work toward precision feeding and individualized animal management. Being easy to use, BCS is the most commonly used energy balance marker by farmers, but a low BCS is only recorded until after negative energy balance is well established and is not negative energy balance specific. In early lactation, BCS can be insufficient to identify if the slimming is normal or excessive. The stakeholders most commonly used indicators of negative energy balance status are milk protein and fat concentrations and fat: protein ratio (Pénasse et al., 2019), which vary within few a days after feeding variation but are also subject to several physiological and environmental variations. In blood, metabolites such as NEFA, BHBA, and glucose are used as negative energy balance biomarkers. NEFA and BHBA are good indicators of body reserve mobilization. NEFA concentration is often measured before calving to identify animals susceptible to ketosis. BHBA, which is the most stable ketone body, is highly reliable only in early lactation. Even if glucose plays a key role in energy metabolism, it is a poor indicator of energy status, as gluconeogenesis balances its concentration. Blood hormones such as IGF-1 and insulin are also closely related to negative energy balance and could be used as biomarkers (Andersen et al., 2004; Chelikani et al., 2004; Gross et al., 2011b). These biomarkers, however, involve blood sampling and analysis, which make them more difficult to use on farms. The monitoring of energy status via indicators measured in milk is easier to perform. In recent years, research on reliable energy status biomarkers among milk metabolites has been undertaken. Bjerre-Harpøth et al. (2012) identified milk citrate as a potential robust indicator of FR, and Billa et al. (2020) found that milk glucose and glutamate concentrations had strong correlations with energy balance and classic indicators of metabolic status.

## Conclusions

FR experiments attempt to induce negative energy balance and thus to mimic early lactation imbalance and food shortage events. Nevertheless, multiple studies conducted on dairy cows have revealed that the responses to this stress are highly variable. The effect of an FR protocol may vary depending on the type of restriction, its length and intensity, the stage of lactation in which it takes place and the responsiveness of the individual. Further research is needed to quantitatively determine these effects using meta-analysis approaches. Some variations seem to be more significant during early lactation, when animals are more likely to use their body reserve to maintain their milk production. Longer and more intense restrictions also affect the significance of animal responses, but some molecules can also vary in different directions depending on the restriction severity. Modifications of plasma composition (glucose, NEFA concentrations, insulin, IGF-I, GH, and cortisol) and milk composition (fat, protein, lactose, and minor metabolites) as well as body weight and BCS reduction induced by FR return to control values within 1 to 14 d after refeeding. Nevertheless, epigenetic mechanisms may regulate milk production for subsequent lactation cycles and even for subsequent generations. Current research aims to identify reliable non-invasive biomarkers of energy balance status. The development of accessible tools to monitor these biomarkers in dairy farming is also required to work toward precision feeding and individualized animal management.

## Abbreviations

ACACA: acetyl-CoA carboxylase 1
BAD: Bcl2-associated agonist of cell death
BCS: body condition score
BHBA: β-hydroxybutyrate
CAPN2: calpain 2
CASP3: caspase 3
CSN1S1: alpha-S1-casein
CSN3: kappa-casein
CTSB: cathepsin B
DMI: dry matter intake
DNA: deoxyribonucleic acid
FABP3: fatty acid-binding protein 3
FR: feed restriction
FSH: follicle stimulating hormone
GH: growth hormone
GHR: growth hormone receptors
GPAM: glycerol-3-phosphate acyltransferase 1
IGF-1: insulin-like growth factor I
IGFBP5: insulin like growth factor binding protein 5
LALBA: alpha-lactalbumin
LH: luteinizing hormone
LPL: lipoprotein lipase
MEC: mammary epithelial cell
miRNA: microRNA
MMP: matrix metallopeptidases
NEFA: non-esterified fatty acids
PTEN: phosphatase and tensin homolog
SCD1: acyl-CoA desaturase 1
SLC2A1: solute carrier family 2 member 1
SREBF1: sterol regulatory element-binding protein 1

## Care and Use of Animals

All the authors confirm that the ethical policies of the journal as mentioned in the journal’s author guidelines have been adhered to.

No ethical approval was required as this is a review article with no original research data.
